# Public funding, social change and uterus transplants: a response to commentaries

**DOI:** 10.1136/medethics-2016-103491

**Published:** 2016-09

**Authors:** Stephen Wilkinson, Nicola Jane Williams

**Affiliations:** Department of Politics, Philosophy, and Religion, Lancaster University, Lancaster, UK

**Keywords:** Transplantation

Our paper ‘Should the State Fund Uterus Transplants?’ was recently published as a feature article alongside commentaries by Alghrani, Balayla and Lotz. We would like to thank all three for their insightful and careful analyses and *JME* for providing us with the opportunity to publish in this format. The commentaries were generally favourable and we have little to add regarding the pieces by Alghrani and Balayla. We would however like to take this opportunity to respond to some challenges and questions raised by Lotz.

Our approach has much in common with hers. In particular, we agree that:

- the most important components of parenthood are social in nature, with the desire to gestate or have genetically related children being secondary;
- many harms linked to infertility are caused or exacerbated by social and cultural attitudes which overestimate the importance of gestational and genetic ties;
- prospective medical treatments should not automatically be disqualified from public funding because the harms they treat have primarily social causes.

Nonetheless, Lotz is sceptical about our proposal that uterus transplants (UTx) should be publicly funded if and when it meets the usual standards of safety, efficacy and cost-effectiveness. There appear to be three main reasons for this:

- Reforming law and practice could render adoption a ‘sufficiently good’ alternative to UTx.
- Our paper underestimates the extent to which the harms of infertility are caused by the sociocultural context in which reproduction takes place, and the likelihood of changing this context.
- State provision of UTx might itself undermine efforts to secure positive attitudinal change.

In what follows, we focus mainly on (ii) and (iii) since (i) is heavily dependent on those more fundamental issues. We note from the outset however that we would of course welcome any reforms of adoption law and practice that serve to make it a more attractive alternative to infertility treatment.

Turning now to (ii), as we explain in our paper and Lotz does in hers, the value individuals attach to parenthood (or certain components thereof), the strength and depth of the desire felt by individuals to parent, and the pain that may result from an inability to fulfil this desire should not be considered in isolation from social context. The extent to which having and parenting a child (and attaining the status of parent in a particular way) is deemed by individuals to constitute a central component of their life plan is, to a great extent, determined by collective norms, preferences and priorities. Thus, in our society, where sexist and essentialist messages regarding the nature of women as carers or mothers still prevail, where government policies (in some areas at least) favour families and reward parenthood, where social parenthood absent genetic links (such as adoption or step-parenthood) is undervalued, and where gestation and childbirth is presented as a transformative experience, it is unsurprising that many women view conception, gestation and childrearing as *necessary* components of a good life—and conversely view infertility as seriously harmful and negative.

In a society absent those norms, preferences and priorities that valorise biological parenthood, the desire to attain it, and the extent and significance of the harms that result from thwarting it, would look very different. In moving away from pronatalism, and the fetishisation and geneticisation of biological parenthood, many of the damaging effects of infertility would be ameliorated—perhaps even to such an extent that such harms would (at least in the vast majority of cases) no longer warrant public funding. Thus we both welcome and encourage attempts to challenge these attitudes as part of a larger social project to secure the equality of all persons and to accept and embrace individual difference.

Yet, while we are aware of the potential benefits of widespread socioreproductive change and hold that the creation of such a society is desirable, our paper was not written for such a society. As such its arguments and conclusions do not apply to any society but our own and those closely resembling it. Thus, the critique which suggests that we underestimate the extent to which the desire to avail oneself of assisted reproductive technologies (ARTs), and UTx specifically, is socioculturally grounded is off target. We recognise this, wish it were different, and welcome attempts to change social and cultural attitudes regarding reproduction and parenthood. We also recognise, however, that social change is unlikely to come quickly or easily, and that as such, the harms that women suffer as a result of their infertility in *our society* and at *this time* deserve to be both recognised and ameliorated where possible.

Lotz’ concern that we do not pay sufficient attention to the prospect of socioreproductive change and our paper's place within efforts to secure that change does, however, raise an interesting and perhaps too often neglected methodological question about bioethics. Should work focus on ‘ground level’ policy issues which take social reality as it is, more or less for granted? Or, should a more visionary or revolutionary approach be taken through focus on fundamental social change? This question arises in numerous areas such as organ sale and sex selection where some scholars focus on the production of shorter-term policy proposals for minimising harm and injustice within a flawed legal and social system, while others focus on the removal of the more fundamental social structures that caused the harm and injustice in the first place. Our view is that there is room for both kinds of work and that our field would be impoverished absent either. Thus it seems that maybe in this respect the difference between our position and that of Lotz is less a case of disagreement and more one of working on different levels and doing different but not incompatible things.

This leads on to Lotz’ third critique: that public provision of UTx (and other fertility treatments) may undermine efforts to secure socioreproductive change by lending credibility to and reinforcing existing biases. She notes: ‘the state's provision and designation of a “treatment” as publicly fund-worthy communicates a powerful venerating message regarding its importance’ and ‘the more resource- and risk-intensive that treatment is, the louder is the validation that the condition to be treated is weighty serious and regrettable, and that the proposed treatment benefits are real, significant, and valuable’ (p. 7).

This kind of problem is of course not unique to infertility treatment. Many forms of cosmetic surgery such as the pinnaplasty, breast reconstruction after mastectomy and indeed (as mentioned in our paper) scalp cooling treatments for persons undergoing chemotherapy are often publicly funded. Such treatments provide no direct functional benefit: one can hear just as well with more or uncommonly shaped ear cartilage, one does not gain increased sensation or the ability to breastfeed after breast reconstruction, and one's hair is no better at protecting against the cold than a wig or a hat. Rather, these interventions aim to protect people from the hostile treatment routinely meted out to those who are unable or unwilling to conform to prevailing aesthetic norms.

In these cases, as in the parallel case of infertility, it might be argued that cosmetic interventions should not be offered because doing so lends credibility to and reinforces prevailing aesthetic norms and the attitude that those who fail to conform deserve hostile or discriminatory treatment.

It seems to us however that such arguments are problematic for three reasons. First, one need not subscribe to the view that the prevailing aesthetic norms are correct in order to offer such treatments; providers could simply see things ‘as they are’ but choose to proceed just in order to protect the individual patient from distress in a defective social situation. Second, such treatments can occur *alongside* educative efforts to change societal attitudes until such time that these treatments no longer confer significant benefits on their recipients. Third, in at least some of these cases, sacrificing the immediate interests and needs of those who are suffering *right now*, as part of a much longer-term strategy to effect attitudinal change, seems unduly harsh and demanding, and those patients who are forced to live without treatment may end up paying a very heavy price as part of their (often involuntary) contribution to wider social change.

These points apply with at least equal force to infertility. While infertility treatment has the potential to cause expressive harm, and indeed sometimes does given the way such treatments are marketed, such harms do not necessarily result from state provision. For, provided it is recognised: that the harms of infertility are primarily the result of social conditions; that those who perform such treatments make this clear to recipients, and position such treatments as no more or less desirable than non-technological options such as adoption or remaining childless; and provision takes place alongside educative efforts designed to improve social attitudes regarding infertility, there seems little reason to conclude that funding should not be provided for this reason at this time.

